# UFT plus gemcitabine combination chemotherapy in patients with advanced non-small-cell lung cancer: a multi-institutional phase II trial

**DOI:** 10.1038/sj.bjc.6602781

**Published:** 2005-09-20

**Authors:** Y Ichinose, T Seto, H Semba, K Itoh, Y Inoue, F Tanaka, J Araki, M Tamanoi, H Yamamoto, N Iwamoto

**Affiliations:** 1 Department of Thoracic Oncology, National Kyushu Cancer Center, 3-1-1, Notame, Minami-ku, Fukuoka 811-1395, Japan; 2 Division of Respiratory Diseases, Kumamoto Regional Medical Center, Kumamoto, Japan; 3 Department of Respiratory Medicine, Shin Beppu Hospital, Oita, Japan; 4 Department of Respiratory Medicine, Isahaya Insurance General Hospital, Nagasaki, Japan; 5 Department of Respiratory Medicine, Kumamoto City Hospital, Kumamoto, Japan; 6 Department of Respiratory Medicine, Yamaguchi Central Hospital, Yamaguchi, Japan; 7 Department of Respiratory Medicine, Minamata General Medical Center, Kumamoto, Japan; 8 Department of Respiratory Medicine, Asou Iizuka Hospital, Fukuoka, Japan; 9 Respiratory Organ and Diabetes Center, Saiseikai Kumamoto Hospital, Kumamoto, Japan

**Keywords:** uracil–tegafur, UFT, gemcitabine, NSCLC, elderly

## Abstract

A multi-institutional phase II trial was conducted to evaluate the efficacy and toxicity of combination chemotherapy consisting of gemcitabine and UFT, which is composed of tegafur and uracil, for non-small-cell lung cancer (NSCLC) patients. Patients with advanced NSCLC received an oral administration of UFT (tegafur 200 mg m^−2^) b.i.d. from days 1 to 14 and intravenous injection of gemcitabine 900 mg m^−2^ on days 8 and 15. This treatment was repeated every 4 weeks. A total of 44 patients were enrolled into this trial. The median age of all patients was 74 years, with 23 patients younger than 75 years and 21 patients with 75 years of age or older. A total of 18 patients (41%) achieved a partial response. The median survival time was 13.2 months and the 1-year survival rate was 59%. The most common grade 3–4 toxicity was neutropenia (57%). The frequency of grade 3 nonhaematologic toxicities was less than 5%. In addition, no significant difference in the response, survival or toxicities was observed between the patients younger than and those older than 75 years of age. This combination chemotherapy demonstrated a promising effectiveness and acceptable toxicity in patients with advanced NSCLC, even in patients older than 75 years.

UFT is an oral anticancer agent composed of tegafur and uracil at a 1 : 4 fixed molar ratio (Fujii *et al*, 1979). Although the clinical effectiveness of the single agent against advanced non-small-cell lung cancer (NSCLC) has not been evaluated in adequate sample size (Keicho *et al*, 1986), a recent randomised phase III trial in 984 patients with completely resected stage I adenocarcinoma demonstrated that postoperative adjuvant chemotherapy with UFT significantly prolonged the survival of patients in comparison to observation alone (Kato *et al*, 2004). The combination chemotherapy of UFT plus cisplatin has also been reported to be an effective treatment for advanced NCSLC. The combination chemotherapy consisting of a daily administration of UFT for 2 or 3 weeks and a bolus injection of cisplatin at the mid-cycle of the administration of UFT yields a response rate of 29–38%, and a median survival time of 10–13 months (Ichinose *et al*, 1995, 2000; Saito *et al*, 2001).

Gemcitabine is an active anticancer agent for the treatment of NSCLC. The objective response rates of patients with advanced NCSLC treated with gemcitabine alone and the combination chemotherapy of platinum plus gemcitabine range from 20 to 26% and from 25 to 61%, respectively (Harper, 2003). A median survival time of 8–16 months in patients treated with the combination chemotherapy has been reported (Harper, 2003).

Both 5-FU generated from tegafur in UFT and gemcitabine are antimetabolites but inhibit DNA synthesis via a different pathway. The main mechanism of inhibiting DNA synthesis by 5-FU is due to 5-FU-derived fluorodeoxy monophosphate binding to thymidylate synthase (Madajewicz *et al*, 1984). The incorporation of gemcitabine triphosphate, which is generated from the phosphorylation of gemcitabine by deoxycytidine kinase, into DNA is most likely the major mechanism by which gemcitabine exerts its cytotoxic action (Huang *et al*, 1991). Such different antitumour mechanisms suggest a potential synergism between 5-FU and gemcitabine. This potential synergism has been indeed observed in *in vitro* studies using various cancer cell lines (Schulz *et al*, 1998; Peters *et al*, 2000).

In our prior phase I trial, the combination chemotherapy of UFT plus gemcitabine was found to be feasible (Seto *et al*, 2002). The most appropriate schedule and dosing was 200 mg m^−2^ of UFT b.i.d for 14 consecutive days with 900 mg m^−2^ gemcitabine on days 8 and 15. The main toxicity was haematologic. The overall response rate was 33%, while the rate was 45% in the 13 patients without any prior chemotherapy. With these backgrounds, we conducted a phase II trial of combination chemotherapy using UFT plus gemcitabine. In the present multi-institutional phase II trial, we found the proportion of elderly, especially patients older than 75 years of age, to be high. Therefore, the results of all patients in the phase II trial and the differences between patients younger than 75 years and those 75 years of age or older were evaluated.

## MATERIALS AND METHODS

### Patient eligibility

The patients were eligible for this phase II trial if they had been either cytologically or histologically confirmed to have NSCLC; stage IIIB without any indications for radiotherapy or stage IV; measurable disease; no prior chemotherapy; an Eastern Cooperative Oncology Group (ECOG) performance status (PS) of 0, 1 or 2; a projected life expectancy of at least 3 months. Other eligibility criteria regarding organ functions were as follows: a leucocyte count of 4000–12 000 l^−1^; platelet count of 100 000 *μ*l^−1^ or greater; haemoglobin level of 9 g dl^−1^ or greater; a serum bilirubin level less than 1.5 mg dl^−1^; serum aspartate aminotransferase and alanine aminotransferase levels of twice the upper limit or less; a serum creatinine level of 1.5 mg dl^−1^ or less. For staging, all patients underwent a computed tomography (CT) scan of the thorax, including the upper abdomen, and either brain CT or magnetic resonance images of the brain, and a radioisotopic bone scan were performed for almost all patients.

Any patients who were pregnant or had concomitant serious diseases, a concomitant malignancy, pleural effusion necessitating treatment or symptomatic cerebral involvement were excluded from the study. However, patients whose malignant pleural effusion was controlled by intrapleural hypotonic cisplatin treatment (Ichinose *et al*, 2003) were eligible. Written informed consent was required from all patients and the protocol was approved by the institutional ethics committee of each of the participating institutions. On entrance to the study, the eligibility of patients was checked via facsimile by the central administration office of the Kyushu Yamaguchi Thoracic Oncology Group (Fukuoka).

### Treatment schedule

UFT (tegafur 400 mg m^−2^ day^−1^) in the form of a 100-mg capsule (100 mg of tegafur plus 224 mg of uracil) was given orally in two separate doses, before meals, from days 1 to 14. The dose was rounded up or down to the nearest 100 mg. Most patients received UFT three capsules (tegafur 300 mg and uracil 672 mg) b.i.d. Gemcitabine (900 mg m^−2^) was dissolved in 20 ml of physical saline and then diluted further with physical saline or 5% glucose to a volume of 250 ml. The gemcitabine solution was administered by intravenous drip infusion over 30 min on days 8 and 15. On the day gemcitabine was administered, a complete blood count was performed and the drug was administered only when the leucocyte count was 2000 *μ*l^−1^ or higher and the platelet count was 70 000 or higher *μ*l^−1^. If these requirements were not met, then drug administration was postponed for a maximum of 4 days. The treatment regimen was repeated every 4 weeks and at least two cycles were administered unless disease progression or an unacceptable toxicity occurred. A leucocyte count of 3000 *μ*l^−1^ or greater and the entry eligibility criteria regarding organ functions had to be satisfied to start the next cycle. The doses of gemcitabine were reduced to 800 mg m^−2^ either when grade 4 haematologic toxicities occurred or when the administration of gemcitabine on day 15 was skipped in a prior cycle. The doses were increased to 1000 mg m^−2^ if both nadirs of the leucocyte and platelet counts were more than 3000 and 100 000 *μ*l^−1^, respectively, in a prior cycle.

### Evaluation of response and toxicity

All eligible patients who received any part of the treatment were considered assessable for response and toxicity. The complete blood count, and blood chemistry studies were repeated weekly. The response was assessed based on the chest X-ray or CT scan findings that initially had been used to define tumour extent. The response was evaluated according to the criteria of the World Health Organization (Miller *et al*, 1981). A central radiologic review was performed to determine the eligibility of patients and the response of treatment. Any adverse events were graded according to the National Cancer Institute-Common Toxicity Criteria (NCI-CTC) version 2.0.

### Statistical analysis

The primary end point of this study was to determine the tumour response rate produced with this treatment protocol. Based on the assumption that a response rate of higher than 40% would warrant a further investigation of this combination chemotherapy, and that a rate of below 20% would make such an investigation unnecessary, a sample size of 36 patients was required with an alpha error of 0.05 and a beta error of 0.2. Therefore, the accrual of 40 patients was planned for a 2-year period since several ineligible patients might be identified in the course of the study.

The overall survival of the eligible patients was defined as the time from the start of the treatment until death from any cause and it was estimated by the Kaplan–Meier method. Differences between the proportions were evaluated by the chi-square test. The data were considered to be significant when the *P*-value was 0.05 or less.

## RESULTS

### Patient characteristics

From July 2000 to September 2002, 44 patients were entered into this phase II trial. Since no upper age limit for the eligibility criteria was established, in contrast to other Japanese trials, many elderly patients were included in this trial. The patient characteristics classified by an age of less than 75 years of age or 75 years of age and older are shown in Table 1. There were no statistically significant differences in the proportions regarding gender, performance status, stage, histology or previous treatment between the two (<75 *vs* ⩾75 years) groups. There were 33 patients who were 70 years of age or older.

### Treatment delivery

The median number of treatment cycles for all patients was three as shown in Table 2. In all, 39 (89%) patients received at least two cycles of the treatment. The reasons for terminating the chemotherapy before the second treatment cycle were adverse events in three patients, a progression of dementia in one and patient refusal in one. Although the proportions of the cycles administered between the two-age groups showed no significant difference, 10 (48%) patients in the older age group received five or more cycles of the treatment.

The administration of gemcitabine on day 15 was skipped in 10 (5%) of a total of 196 cycles. A dose decrease to 800 mg m^−2^ and the increase to 1000 mg m^−2^ were reported in four and five patients, respectively.

### Adverse events

The main adverse events were haematologic toxicities as shown in Table 3. Grade 3 and 4 neutropenia was reported in 57% of the patients and grade 4 in 20%, while no grade 4 anemia or thrombocytopenia was observed. The frequency of grade 3 and 4 haematologic toxicities was 57% in the younger-age group and 62% in the older-age group. The frequency of grade 3 or greater nonhaematologic toxicities was 5% or less. No grade 4 nonhaematologic toxicity was observed.

### Response

Among the 44 patients, 18 patients showed a partial response (41%; 95% confidence interval, 26–55%). There were 24 patients (55%) with no change and two patients (5%) with progressive disease. There were no differences in the response rate according to age (<75 *vs* ⩾75 years, 44 *vs* 38%), gender (male *vs* female, 46 *vs* 33%), stage (III *vs* IV, 42 *vs* 41%) and histology (adenocarcinoma *vs* others, 44 *vs* 33%). Median duration of response was 6.9 months.

### Survival

**Figure 1:**
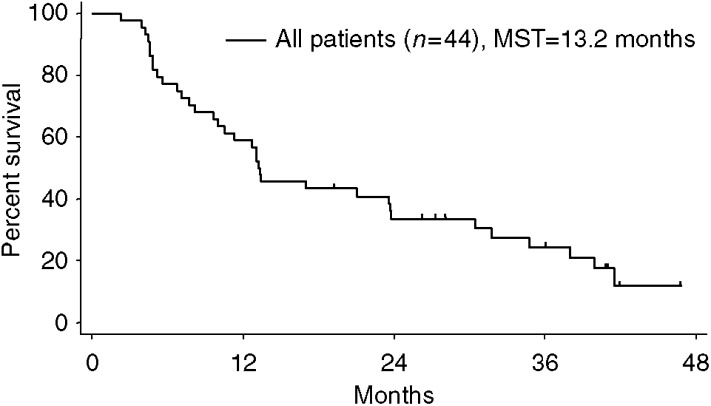
Overall survival. Each tick represents a patient who is alive.

The overall median follow-up time for all patients was 38 months (range from 23 to 50 months). As shown in Figure 1, the median survival time of all 44 patients was 13.2 months, and the survival rates at 1 and 2 years were 59% (95% confidence interval, 45–74%) and 34% (95% confidence interval, 20–48%), respectively. There were no statistically significant differences in survival between the patients under 75 and those over 75 years (*P*=0.4948). The median survival time was 13.2 and 13.3 months, respectively.

## DISCUSSION

Platinum-based combination chemotherapy is recommended for the treatment of advanced NSCLC patients with a good performance status (Pfister *et al*, 2004). However, the incidence of severe adverse effects induced by this combination chemotherapy is indeed more frequently observed in elderly patients than younger patients even if the subjects have good performance status (Langer *et al*, 2002). Therefore, a single agent such as vinorelbine, whose effect on prolonging survival in advanced NSCLC with elderly has been demonstrated in comparison to the best supportive care (Anonymous, 1999), is sometimes selected to treat such patients in practice. In addition, the combination of vinorelbine plus gemcitabine has been reported to be not more effective than single-agent vinorelbine or gemcitabine in the treatment of elderly patients with advanced NSCLC (Gridelli *et al*, 2003).

Lung cancer is primarily a disease of the elderly. More than one half of new diagnoses and more than two-thirds of annual deaths occur in patients over 65 years (Havlik *et al*, 1994; Gridelli *et al*, 1997). Although the aim of the present study was not to develop a treatment for only the elderly, the median age of the patients in this study was 74 years and 48% of the patients were 75 years or older. The reasons for this age distribution were thought to be partly due to the followings: First, the subjects of almost all previous clinical trials for chemotherapy against advanced NSCLC conducted in Japan were limited to patients under 75 years of age. Second, both UFT and gemcitabine were considered to be relatively safe anticancer agents. Third, they could be administered on an outpatient basis with no need for premedication and prehydration.

The overall response rate of the present trial using UFT and gemcitabine was 41%. In a phase I trial of this combination chemotherapy, the response rate of patients without prior chemotherapy was also reported to be 45% (Seto *et al*, 2002). This high antitumour effect may lend support to the sequence of administration of UFT and gemcitabine. In fact, an *in vitro* study has shown the sequence-dependent antitumour effects of the combination of 5-FU and gemcitabine to be seen with a maximum effect when 5-FU preceded gemcitabine (Rauchwerger *et al*, 2000). Since the 5-FU concentration in blood reaches a steady state 5 days after the start of UFT administration (Ho *et al*, 1998), the administration of gemcitabine on days 8 and 15 is considered to be most appropriate.

Since the recent randomised trial demonstrated that gemcitabine plus carboplatin produced a significantly higher response rate and survival rate than gemcitabine alone in advanced NSCLC patients including a substantial proportion of elderly patients (37% of patients >70 years old), the combination chemotherapy using gemcitabine and carboplatin (Sederholm, 2002) may also be considered to be a suitable chemotherapy regimen for the treatment of elderly patients. The response rate and median survival time in the combination chemotherapy group has been reported to be 30% and 10 months, respectively. Grade 3 or 4 nonhaematologic toxicity was observed in 26% of the patients, while grade 3 and 4 thrombocytopenia was reported in 24% each of patients, respectively. A toxicity profile in the elderly has not yet been reported.

In the present phase II trial of 44 patients with a median age of 74 years, including 21 patients who were 75 years or older, the response rate was 41% and the median survival time was 13.4 months. These observations suggest that this combination chemotherapy is also worthy of further investigation for the treatment of all NSCLC patients including the elderly. In addition, it should be confirmed whether or not this combination regimen is equally effective in ethnic groups other than Japanese.

## Figures and Tables

**Table 1:** Patient characteristics

	**<75 years (*n*=23)**	**⩾75 years (*n*=21)**	**Total (*n*=44)**
*Age*			
Median (range)	70 (58–74)	78 (75–89)	74 (58–89)
			
*Gender*			
Male/female	7/16	11/10	18/26
			
*Performance status (ECOG)*			
0/1/2	8/13/2	8/13/0	16/26/2
			
*Stage*			
III/IV	5/18	7/14	12/32
			
*Histology*			
Adenoca/others	17/6	15/6	32/12
			
*Previous treatment*			
None	11	11	22
HPT^a^	6	8	14
Operation	5	2	7
Radiotherapy	1	0	1

a Hypotonic cisplatin treatment.

ECOG=Eastern Cooperative Oncology Group.

**Table 2:** Treatment delivery

**Cycle of treatment**	**<75 years (*n*=23)**	**⩾75 years (*n*=21)**	**Total (*n*=44)**
1	23 (100%)	21(100%)	44 (100%)
2	21 (91%)	18 (86%)	39 (89%)
3	12 (52%)	14 (67%)	26 (59%)
4	9 (39%)	11 (52%)	20 (45%)
⩾5	6 (26%)	10 (48%)	16 (36%)
			
*No. of cycles*			
Median	3	4	3
Range	1–18	1–16	1–18

**Table 3:** Haematologic and nonhaematologic toxicities (*n*=44)

	**Grade**				
**Toxicity**	**1**	**2**	**3**	**4**	**Frequency of Grade 3 or 4 (%)**
Leukopenia	4	19	13	1	32
Neutropenia	5	8	16	9	57
Anemia	22	13	4	0	9
Thrombocytopenia	15	9	8	0	18
GOT	8	1	0	0	
GPT	10	1	0	0	
Creatinine	1	0	0	0	
Anorexia	4	1	2	0	5
Vomiting	2	2	0	0	
Diarrhoea	0	1	2	0	5
AIP	0	0	1	0	2
Febrile neutropenia	0	0	1	0	2

GOT=glutamic oxaloacetic transaminase; GPT=glutamic pyruvic transaminase; ALP=alkaline phosphatase.
